# Engineering *Lactococcus cremoris* strains co-expressing two cellulase genes for growth on cellulose

**DOI:** 10.3389/fbioe.2026.1857763

**Published:** 2026-06-17

**Authors:** Petra Štravs, Henri-Pierre Fierobe, Stéphanie Perret, Aleš Berlec

**Affiliations:** 1 Department of Biotechnology, Jožef Stefan Institute, Ljubljana, Slovenia; 2 Interdisciplinary Doctoral Study Program in Biosciences, University of Ljubljana, Ljubljana, Slovenia; 3 Aix-Marseille Université, CNRS, LCB-UMR7283, Marseille, France; 4 Faculty of Pharmacy, University of Ljubljana, Ljubljana, Slovenia

**Keywords:** Cel5H, Cel5I, Cel9A, co-expression, endoglucanases, lactic acid bacteria, cellulose, synergy

## Abstract

**Introduction:**

Lactic acid bacteria are commonly used to convert carbohydrates into valuable products like lactic acid, but they currently rely on carbon sources from food crops, raising sustainability and cost concerns. Lignocellulosic waste is a more sustainable alternative, yet these bacteria cannot naturally break down cellulose to grow on it. Efficient cellulose degradation requires synergetic action of multiple cellulolytic enzymes, each operating through distinct mechanisms.

**Methods:**

To design genetic constructs for co-expression of gene pairs encoding different heterologous cellulases (Cel5I, Cel5H or Cel9A) from separate expression cassettes in *Lactococcus cremoris*, the BglBrick approach was used. The activity of culture supernatants containing co-expressed cellulases was assessed on microcrystalline cellulose and phosphoric acid swollen cellulose (PASC), and compared with that of co-cultures and cultures expressing individual enzymes. Subsequently, growth of selected developed strains on PASC was evaluated.

**Results:**

The A1 strain that co-produced cellulase pair Cel5H-Cel9A achieved the highest activity on PASC and one of the highest activities measured on microcrystalline cellulose. Additionally, the A1 strain showed the highest degradation of PASC during the 16 days of anaerobic cultivation and one of the highest lactic acid production levels.

**Discussion:**

In this study, co-expression of gene pairs that encode different heterologous cellulases from separate promoters in *L. cremoris* was achieved for the first time. This approach allows each cellulase to be tuned independently, enabling optimization of their expression levels to maximize synergistic effects and enhance the cellulolytic performance of the engineered bacteria.

## 1 Introduction

Industries ranging from wood processing, paper manufacturing to food processing and agriculture, along with households, generate large amounts of lignocellulosic waste. Instead of being discarded, these waste materials can serve as a low-cost source of carbon, offering a sustainable alternative to the traditional substrates, derived from edible and energy crops such as corn, sugar beet and sugar cane, to be used in microbial fermentations (Kim et al., 2022). Cellulose represents the dominant polymer within lignocellulosic biomass. To unlock the full potential of cellulose as a source of glucose for microbial processes, a combination of cellulolytic enzymes with distinct cellulose-degrading modes, working in synergy, are required (Liu et al., 2021). Cellulases can degrade cellulose either in a processive or non-processive way. Processive cellulases hydrolyse cellulose sequentially by sliding along a cellulose chain without dissociating, whereas non-processive cellulases dissociate after each or a few cleavage events (Wu and Wu, 2020). In addition, cellulases can be classified according to where they act on the substrate. Endocellulases cleave β-1,4-glycosidic bonds at random internal positions within cellulose chains, generating cellodextrins of varying length, while exoglucanases primarily release cellobiose units from the reducing or non-reducing ends of cellulose chains. In the final step of cellulose hydrolysis, β-glucosidases convert cellobiose into glucose monomers, which can subsequently enter glycolysis and be utilized by microorganisms (Liu et al., 2021).

Lactic acid bacteria (LAB) are versatile microbial cell factories capable of converting carbohydrates into valuable biochemicals such as lactic acid (Yankov, 2022). Apart from lactic acid, LAB are also known producers of vitamins, flavour compounds, bacteriocins and exopolysaccharides (Mazzoli et al., 2014). Although no naturally occurring LAB have been shown to grow independently on cellulose due to the lack of efficient cellulolytic machinery (Zabidi et al., 2020; Román Naranjo et al., 2022; Chatgasem et al., 2023), several studies reported the ability of LAB to grow on degradation products of cellulose (cellodextrins, e.g., cellobiose, cellotriose, cellotetraose) (Okano et al., 2010; Gandini et al., 2017; Zhang et al., 2019). This makes LAB ideal candidates for engineering cellulolytic ability in this group of industrially relevant microorganisms. Several attempts have been made to produce single cellulases or co-produce cellulase pairs in LAB. These studies evaluated: (i) the cellulolytic properties of LAB by measuring only the activity of heterologously produced cellulases and cellulolytic multienzyme complexes (Moraïs et al., 2013; Moraïs et al., 2014; Stern et al., 2018), (ii) silage ensiling capabilities of designed LAB (Guo et al., 2019; Liu et al., 2019; Wan et al., 2024; Ma et al., 2025), or (iii) ability of LAB to grow on soluble cellodextrins (Gandini et al., 2017; Zhang et al., 2019). However, there are almost no reports on LAB growth or viability and ability to degrade cellulose when cellulose is used as the main carbon source (Štravs et al., 2025). As co-production of cellulases may lead to toxicity, numerous studies addressed this limitation by establishing co-culture systems in which individual strains produce a single cellulase or a single enzymatic unit of a multienzyme complex (Liu et al., 2019; Moraïs et al., 2014; Stern et al., 2018; Wan et al., 2024; Ma et al., 2025).

A recent study showed for the first time that engineered *Lactococcus cremoris* cells producing a single heterologous cellulase (Cel9A, Cel5I or Cel5H) can grow on amorphous cellulose as the main carbon source (Štravs et al., 2025). Cel9A, Cel5I, and Cel5H were selected for heterologous expression in *L. cremoris* NZ9000 because they are highly active cellulases that play key roles in cellulose degradation and growth of their native cellulolytic microorganisms (*Lachnoclostridium phytofermentans*, *Ruminiclostridium cellulolyticum*, *Saccharophagus degradans* receptively) (Franche et al., 2016; Vita et al., 2019; Watson et al., 2009; Tolonen et al., 2009). In addition, all three enzymes produce cellobiose as the main degradation product, which can be metabolized by *L. cremoris* NZ9000. Building on these results, the present study sought to co-produce the corresponding cellulases (Cel9A, Cel5I or Cel5H) in *L. cremoris* cells, to exploit potentially synergistic activity of these cellulases. Although all three cellulases are endoglucanases, they act with different modes of action (Cel9A and Cel5H are processive endoglucanases, while Cel5I is non-processive endoglucanase) (Zhang et al., 2010; Watson et al., 2009; Franche et al., 2016), which indicates their potential cooperativity. Bacteria that lack exoglucanases (cellobiohydrolases) in their cellulolytic systems exist (e.g., *Saccharophagus degradans*) and rely solely on processive endoglucanases (Watson et al., 2009; Zhang et al., 2014). These enzymes are functionally equivalent to a combination of non-processive endoglucanases and exoglucanases, which, together with β-glucosidases (that breaks down cellobiose to glucose), enable growth of these organisms on cellulosic substrates (Watson et al., 2009). It is known that *L. cremoris* NZ9000 possesses active endogenous intracellular β-glucosidases and cellobiose-specific phosphotransferase transporter; enabling the strain to utilise cellobiose as carbon source (Linares et al., 2010).

In this study, genetically engineered *L. cremoris* strains co-expressing two cellulase genes from separate promoters were developed. Cellulolytic activities on amorphous and microcrystalline cellulose were compared between (i) *L. cremoris* monocultures simultaneously producing two different cellulases, (ii) *L. cremoris* co-cultures, with each strain producing a different cellulase, and (iii) *L. cremoris* monocultures producing a single cellulase. In addition, anaerobic growth of the most promising *L. cremoris* strains on amorphous cellulose as a substrate was evaluated, and cell viability, production of fermentation metabolites and total degradation of the cellulosic substrate were monitored.

## 2 Materials and methods

### 2.1 Assembly of expression plasmids

Target nucleotide sequences were amplified with repliQa HiFi DNA Polymerase (Quantabio) using specific primers (IDT) that are listed in the (Supplementary Table S1). The obtained amplicons were digested with FastDigest restriction enzymes (Thermo Scientific) and ligated into plasmid backbones (Supplementary Table S1) with T4 DNA ligase (New England Biolabs). Purification of DNA fragments was performed using the NucleoSpin Gel and PCR Clean-up Kit (Macherey-Nagel). To obtain expression cassettes for inducible expression of cellulase genes *cel5I*, *cel9A* and *cel5H* (NCBI accession numbers: WP_012634873.1, WP_312101035.1 and WP_011469710.1, respectively), PnisA promoter was amplified from pNZ8148 plasmid and used to replace the constitutive promoter PepN between the restriction sites *Bgl*II/*Nco*I in plasmids pPepN_sp_Cel9A, pPepN_sp_Cel5H and pPepN_sp_Cel5I from Štravs et al. (2025). Expression cassettes containing promoter (PnisA or PepN), gene encoding cellulase (*cel5I*, *cel9A* or *cel5H*) with N-terminal secretion signal peptide spUsp45 (derived from the endogenous Usp45 protein), and transcription terminator were amplified from plasmids pPepN_sp_Cel9A, pPepN_sp_Cel5H, pPepN_sp_Cel5I, pPnisA_sp_Cel9A, pPnisA_sp_Cel5H and pPnisA_sp_Cel5I, using primer pairs NB-F-PpepN and BX-R-TT2 or NB-F-PnisA2 and BX-R-TT2 and inserted into pNBBX plasmid between *Bgl*II/*Xho*I restriction sites. Insertion of the second expression cassette into the pNBBX plasmids was performed using BglBrick approach according to Plavec et al. (2021). List of plasmids assembled in this study is provided in Supplementary Table S1. The cloning was carried out in *L. cremoris* NZ9000 strain. Bacterial cells were transformed by electroporation as described previously (Holo and Nes, 1995), using a BTX Gemini X2 system. Plasmid isolation was conducted with the NucleoSpin Plasmid EasyPure kit (Macherey-Nagel) including an additional 30 min incubation with mutanolysin (62.5 U/mL) and lysozyme (195,000 U/mL). The verification of assembled plasmids was performed by DNA sequencing (Eurofins).

### 2.2 Bacterial growth and gene expression

*Lactococcus cremoris* was cultured in M17 medium (Millipore) with 5 g/L glucose (Formedium) at 30 °C under microaerophilic, non-agitated conditions. Plasmid-containing strains were maintained with 10 μg/mL chloramphenicol (Sigma-Aldrich). For analysis of heterologous cellulase synthesis in *L. cremoris* cells, overnight cultures were diluted 50-fold into fresh medium. Cellulase gene expression from the nisin-inducible promoter PnisA was induced with 25 ng/mL nisin (Fluka) at the time of inoculation. Overnight cultures were collected and used for further analysis.

### 2.3 SDS-PAGE of conditioned medium

Overnight cultures with induced expression of cellulase genes were centrifuged (10 min, 4 °C, 4,860 × *g*) to separate conditioned medium from the cells. Proteins in the conditioned medium were precipitated with 10% (w/v) TCA (Sigma-Aldrich), washed with ice-cold acetone, and resuspended in 5× Laemmli buffer, where the volume of 5× Laemmli buffer was 180 times lower than the initial volume of conditioned medium. Prior to SDS-PAGE, dithiothreitol (DTT) (Thermo Fisher Scientific) was added, and samples were boiled for 10 min at 100 °C. Stain-Free SDS-PAGE gels (10%; Bio-Rad) were used for separation of secreted proteins. Separation was carried at 35 mA. Gels were UV-photoactivated for 1 min and imaged using the ChemiDoc MP system with ImageLab v5.1. Molecular weights were estimated using Precision Plus Protein™ All Blue Standard (Bio-Rad). Densitometric analysis of gel images was carried out using GelAnalyzer software version 19.1. Relative intensity was calculated from the intensity of the band corresponding to a cellulase and divided by the intensity of the band corresponding to the major extracellular protein Usp45.

### 2.4 Zymography

Overnight *L. cremoris* cultures with expressed cellulase genes were centrifuged (10 min, 4 °C, 4,860 × *g*) and supernatants (clarified medium) were then concentrated (20×) using Pall’s concentrators with 10 kDa molecular weight cut-off (MWCO). Zymography was conducted as previously described by Cai et al. (2010) with modifications. Samples were mixed with Laemmli buffer containing DTT. Proteins were separated on 10% SDS-PAGE gels at 25 mA, with electrophoretic chamber placed in ice. After electrophoresis, the 10% SDS-PAGE gel with separated proteins was incubated for 2 h at room temperature in renaturation buffer (25 mM Tris-HCl (pH 7.0), 0.1% Triton X-100) with gentle agitation. Buffer was exchanged three times. After renaturation, the polyacrylamide gel was covered with a thin layer of 0.5% agarose gel containing 0.1% carboxymethyl cellulose (CMC) (Sigma-Aldrich) and incubated for 6 h at 30 °C. The nondegraded CMC in agarose layer was then stained with 0.1% Congo red solution (Sigma-Aldrich) for 15 min, followed by washing with 1 M NaCl. In parallel, proteins in samples were separated also on Stain Free gels for later protein visualization.

### 2.5 Activity of cellulases on microcrystalline and amorphous cellulose

The activity of secreted cellulases in the medium was assessed on microcrystalline cellulose Avicel (Supelco) or on phosphoric acid–swollen cellulose (PASC) by measuring the amount of released reducing sugars using the ferricyanide assay described by Park and Johnson (1949). Phosphoric acid–swollen cellulose (PASC) was prepared from Avicel according to the protocol of Wood (1988), and samples containing secreted cellulases for activity assays were prepared as previously described (Štravs et al., 2025). Briefly, overnight cultures were centrifuged and the resulting supernatants were exchanged into 50 mM potassium phosphate buffer (pH 7.0) and concentrated 20-fold with Vivaspin concentrators (10 kDa cutoff). Subsequently, 150 µL of concentrate was added to 3 mL substrate solution (3.5 g/L Avicel or PASC in 50 mM potassium phosphate buffer) and incubated at 30 °C, 70 rpm for 24 h. The concentrations of released soluble reducing sugars during cellulolytic activity on cellulosic substrates were expressed as glucose equivalents.

### 2.6 Anaerobic growth of genetically engineered *L. cremoris* on PASC

Overnight cultures of *L. cremoris* secreting heterologous cellulase were inoculated (1:50) into 45 mL of M17 medium containing 1.5 g/L PASC and 40 μg/mL thiamphenicol. Cultures were grown anaerobically under argon atmosphere for 16 days at 32 °C. Growth, viability and cellulolytic activity on PASC were assessed by determining colony forming units (CFU), metabolic acid production, and amount of residual cellulose, as described below. At four-day intervals, 500 µL samples were collected and centrifuged at 18,000 × *g* for 10 min to separate supernatants and pellets.

### 2.7 Quantification of residual cellulose after cultivation on PASC

At the end of cultivation, residual cellulose present in the pellets of 500 µL culture samples was first acid hydrolyzed with 12 M H_2_SO_4_ for 1 h at 37 °C, followed a 12-fold dilution in distilled water and subsequent thermal treatment (120 °C, 1 h) to generate single glucose units. The obtained glucose was quantified by high-performance anion-exchange chromatography with pulsed amperometric detection (HPAEC-PAD) using a Dionex ICS 3000 system (Sunnyvale) with (4 × 250 mm) CarboPac PA1 column (Fosses et al., 2017).

### 2.8 Profiling of fermentation metabolites formed during growth on PASC

To quantify fermentation products lactate, formate, acetate and ethanol produced during *L. cremoris* growth, 200 µL of culture supernatants were mixed with 50 µL of 25 mM H_2_SO_4_, and analyzed with HPLC on an Aminex HPX-87H column as previously described (Borne et al., 2020). Product concentrations were determined using standards of known concentrations.

### 2.9 Viability assay

To evaluate the number of viable cells of *L. cremoris* during growth on PASC, the number of colony-forming units per milliliter (CFU/mL) was determined. Serial tenfold dilutions of cell culture samples were prepared in sterile PBS, and 10 µL aliquots from each dilution were plated on M17 agar containing 5 g/L glucose. To quantify all viable cells and compare them to the viable cells that harbored the plasmid, the cells were plated on GM17 agar plates either with or without chloramphenicol (10 μg/mL). The plates were incubated overnight at 30 °C.

### 2.10 Statistical analysis

Results are expressed as mean values ±standard deviation (SD) from at least three independent biological replicates as described. Statistical analyses were conducted using GraphPad Prism (version 10.00). Group comparisons were performed using one-way ANOVA with appropriate multiple-comparison *post hoc* tests. Statistical significance was accepted at p < 0.05.

## 3 Results

### 3.1 Engineering of *L. cremoris* for co-production and secretion of two heterologous cellulases

**FIGURE 1:**
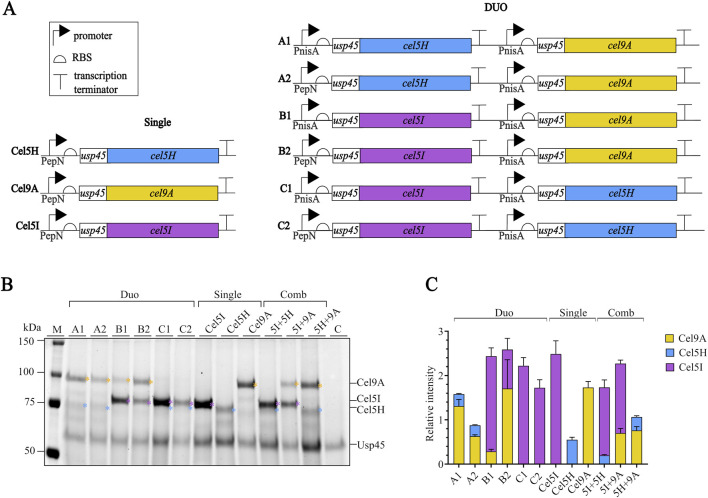
Expression of one or co-expression of two cellulase genes in *L. cremoris* cells. **(A)** Constructs designed for expression of one cellulase gene (*cel5H*, *cel9A*, *cel5I*) or simultaneous expression of two cellulase genes (A1, A2, B1, B2, C1, C2). usp45: signal peptide for protein secretion. PepN: constitutive promoter. PnisA: nisin-inducible promoter. **(B)** SDS-PAGE of TCA-precipitated conditioned media, of *L. cremoris* cultures secreting the cellulases. **(C)** Densitometric analysis of cellulase bands on SDS-PAGE gel from **(B)**. Values represent the mean ± standard deviation (SD) obtained from three independent biological replicates. Single: *L. cremoris* cells expressing one cellulase gene (*cel5H*, *cel9A*, *cel5I*). Duo: *L. cremoris* cells simultaneously expressing two cellulase genes (A1, A2, B1, B2, C1, C2). Comb: co-cultures of two *L. cremoris* strains each expressing one cellulase gene. M: protein molecular weight marker. C: *L. cremoris* cells transformed with empty pNBBX plasmid. The blue, pink and yellow asterisks on the SDS-PAGE indicate cellulases Cel5H, Cel5I and Cel9A, respectively.

The constructs for co-expression of two different cellulase genes were designed so that each gene was transcribed from a separate promoter (Figure 1A). Since construction of plasmids for co-expression of two cellulase genes from two constitutive promoters in *L. cremoris* was not successful, possibly due to the toxicity of overproduced cellulases, two inducible promoters PnisA, or combination of inducible promoter PnisA and constitutive promoter PepN were used. PnisA promoter, induced with 25 ng/mL nisin, and constitutive PepN promoter were previously shown to yield comparable levels of cellulase Cel9A (Štravs et al., 2025). Production of each cellulase in cells that simultaneously produced two enzymes was evaluated with SDS-PAGE of concentrated conditioned medium and compared with the production profiles obtained from monocultures producing a single cellulase or from co-cultures of two single-cellulase producing strains (Figures 1B,C). Cellulases were successfully produced in most strains that were obtained, except for strains C1 and C2, where production of cellulase Cel5H was below the level of detection. Production of each cellulase was the highest when produced alone in monoculture. Conversely, production of each cellulase in co-culture was reduced approximately by half, indicating equal growth of the two strains inoculated at a 1:1 ratio. For some promoter combinations, co-expression of genes for two different cellulases resulted in higher total quantity of cellulases compared to when the corresponding cellulases were produced in co-cultures (Figures 1B,C). Co-expression of two cellulase genes from different promoters (PnisA and PepN) affected the ratios of produced cellulases compared to their co-expression from identical promoters (Figures 1B,C). Production of cellulase Cel5H was similar regardless of promoter (PnisA or PepN in constructs A1 and A2, respectively). However, the type of promoter used for Cel5H markedly affected the co-production of the second cellulase (Cel9A), although the expression of its gene was driven by the same promoter (PnisA) in both constructs. In contrast, production of cellulase Cel5I was substantially increased when its gene was expressed from PnisA promoter in construct B1, relative to the expression from PepN promoter in construct B2. Correspondingly, the production level of the second cellulase, Cel9A, was reduced when expressed from construct B1 compared to its production when expressed from construct B2.

### 3.2 Activity of cellulase combinations on different cellulose substrates

**FIGURE 2:**
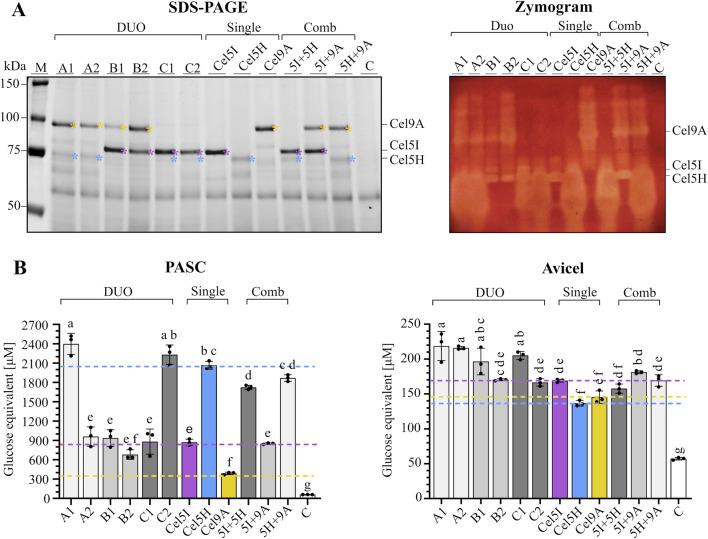
Cellulase activity in overnight culture medium containing cellulases secreted by different genetically engineered *L. cremoris* strains. **(A)** SDS-PAGE of concentrated conditioned media (left) and corresponding zymogram (right). **(B)** Reducing sugars released from PASC (left) and from Avicel (right) during cellulolytic activity. Duo: medium of *L. cremoris* cells simultaneously expressing two cellulase genes (A1, A2, B1, B2, C1, C2). Comb: medium of co-cultures of 2 *L. cremoris* strains each expressing one cellulase gene. C: medium of *L. cremoris* cells transformed with empty pNBBX plasmid. M: protein molecular weight marker. Blue, pink and yellow dashed lines are showing activity reached by single expressed cellulase (Cel5H, Cel5I and Cel9A, respectively) in monoculture. Data are presented as mean ± SD from three biological replicates. Statistical significance is presented with letters (a-g) above the error bars. Samples that do not share a letter are significantly different from each other (one-way ANOVA, p < 0.05).

To assess whether cellulases are active when co-produced in pairs within a single strain, or in co-cultures of two strains, we employed zymography to detect the cellulolytic proteins in concentrated culture medium, using carboxymethylcellulose (CMC) as substrate (Figure 2A). We confirmed the activity of both cellulases in cellulase pairs Cel5H-Cel9A and Cel5I-Cel9A, either when co-produced in a single strain or when produced in co-cultures of strains producing single cellulase. Since cellulases Cel5H and Cel5I have similar molecular weight, and activity of Cel5H cellulase on CMC is the highest among all three cellulases (Štravs et al., 2025), the band corresponding to Cel5H cellulase presumably overlaid the band produced by Cel5I cellulase. Using zymography, we also confirmed successful co-production of Cel5H cellulase in constructs C1 and C2 that was not detected on SDS-PAGE gel (Figures 1B,C). Cellulolytic activity of dialyzed culture media from different cellulase production systems (co-culture, monoculture, co-production, individual production) was also evaluated on microcrystalline (Avicel) and amorphous cellulose (PASC) to see if cellulases, present in different pairs, quantities and ratios, exert synergistic activity on different cellulosic substrates (Figure 2B).

The quantities of each cellulase in the corresponding cellulase pairs (estimated from SDS-PAGE gels) indicate that co-produced cellulase pairs Cel5H-Cel9A and Cel5H-Cel5I could reflect synergistic activity on PASC. The joint measured activity of these cellulase pairs was higher than the sum of their normalized theoretical activities (determined on the basis of measured activities of the corresponding individual cellulases normalized to their quantities) (Supplementary Figure S1). Potential synergism was observed only for co-cultures producing these cellulase combinations, or strains A1 (co-producing Cel5H-Cel9A) and C2 (co-producing Cel5H-Cel5I). Of note, the cellulases produced by strains A2 and C1, which also co-produced these two cellulase pairs (Cel5H-Cel9A and Cel5H-Cel5I, respectively), did not show notable synergy. As these two strains produced different ratios of the corresponding cellulases relative to the synergistic strains A1 and C2 (due to different promoters used, Figure 1), it appears that the ratio between the cellulases as well as the quantity of each cellulase are important for their synergistic activity.

Although potential synergy was suggested for different cellulase combinations on PASC, only the combination Cel5H-Cel9A produced by the A1 strain exhibited significantly higher activity than Cel5H (16%), the single cellulase with the highest activity on amorphous cellulose of all the single cellulases tested (Figure 2B). The synergistic cellulase pairs produced by other strains showed comparable (C2 strain) or lower (Cel5H-Cel9A and Cel5H-Cel5I co-cultures) total activity than Cel5H, most likely due to the lower amount of each cellulase when co-produced or produced in co-culture, compared to the expression of a single corresponding cellulase in monoculture. Potential synergistic activities were also indicated on Avicel, mainly for the co-produced Cel5H-Cel9A and Cel5H-Cel5I pairs, same as was observed on PASC (Supplementary Figure S1). While co-production of two cellulases from constructs A1, A2 and C1 resulted in significantly higher activity on Avicel compared to single-cellulase production or co-cultures of the same enzymes (20%–30%) (Figure 2B), the overall activity on microcrystalline cellulose was still substantially lower than the activity observed on amorphous cellulose.

### 3.3 Growth of *L. cremoris* strains on PASC

**FIGURE 3:**
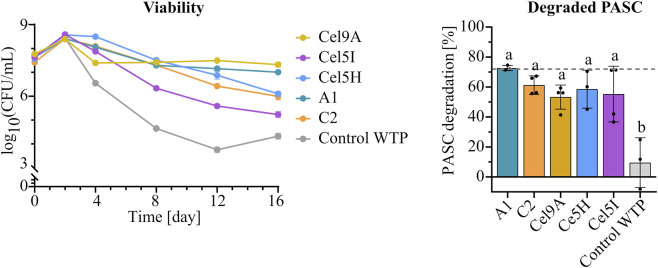
Viability and cellulolytic activity of *L. cremoris* strains expressing different cellulases during anaerobic growth on cellulose. Left: amount of viable *L. cremoris* cells in culture medium during growth. Right: percentage of PASC degraded by each *L. cremoris* strain. The glucose concentration measured for non-inoculated medium was set as 100% of the PASC present in the culture medium. Cel9A, Cel5I, Cel5H: *L. cremoris* NZ9000 expressing a single corresponding cellulase gene. A1: *L. cremoris* NZ9000 co-expressing *cel5H* and *cel9A* cellulase genes. C2: *L. cremoris* NZ9000 strain co-expressing *cel5H* and *cel5I* cellulase genes. Control: *L. cremoris* NZ9000 carrying an empty pNBBX plasmid grown in M17 medium supplemented with PASC (WTP). Data are presented as mean ± SD from at least three biological replicates. Statistical significance is presented with letters (a, b) above the error bars. Samples that do not share a letter are significantly different from each other (one-way ANOVA, p < 0.05).

To evaluate the ability of genetically engineered *L. cremoris* strains to grow on cellulose, the strains were cultivated under anaerobic conditions in media supplemented with PASC as sole carbon source. Only PASC was used to evaluate growth as degradation of Avicel was less efficient (Figures 2A,B). As the strains A1 and C2 (producing cellulase combinations Cel5H-Cel9A and Cel5H-Cel5I, respectively) showed highest average activity on PASC (Figures 2A,B), these two strains were selected for growth experiments. The growth of A1 and C2 strains on PASC was compared to that of wild type strain and the strains that produced only a single heterologous cellulase. Throughout the growth on PASC, we monitored cell viability and plasmid stability, and we determined the final percentage of degraded PASC. Viability was assessed by determining the number of colony forming units per milliliter of culture (CFU/mL). Plasmid maintenance was evaluated by comparing the number of colonies grown on agar plates with and without chloramphenicol, thereby allowing estimation of the proportion of viable cells carrying the plasmid for heterologous expression of cellulase genes. All recombinant cellulase producing strains maintained notably higher viability than the wild type strain, whose viable cell count dropped gradually for approximately 4.5 logarithmic units in 14 days (Figure 3). The cultures of strains Cel9A and A1 contained the highest number of viable cells at the end of cultivation (after 16 days of growth). In all strains, the viability stopped increasing between day 2 and day 4 of cultivation. Strain Cel9A in particular showed the largest decrease (one logarithmic unit) in cell viability on day 4 among all strains expressing cellulases. Nevertheless, the viability of this strain after the day 4 remained unchanged until the end of cultivation, while the viability of all other strains decreased. This might be explained by different PASC degradation products obtained by different cellulases (Supplementary Figure S3) and different ability of *L. cremoris* NZ9000 strain to metabolize these products (Supplementary Figure S4). Almost all selected cellulases and their combinations degrade PASC to cellobiose and cellotriose which can be both utilized by *L. cremoris* NZ9000. After 1 day of incubation with PASC, the cellulase Cel9A was the only cellulase that produced mainly cellotetraose, with only 30% of all products being cellotriose and cellobiose (Supplementary Figure S3). Cellotetraose cannot be metabolized by *L. cremoris* NZ9000 until it is further degraded to cellotriose, cellobiose and glucose. An insufficient and slow supply of metabolizable degradation products might explain the sudden decline in viability of the Cel9A strain after 4 days of cultivation on PASC. On the other hand, only the Cel5H strain retained maximal activity till fourth day of cultivation. Importantly, Cel5H cellulase showed the highest activity on PASC, and produced the highest total amount of degradation products that can be utilized by *L. cremoris* NZ9000 (Figure 2B; Supplementary Figure S3), thereby maintaining its viability on day 4. Stable plasmid maintenance for heterologous cellulase gene expression was confirmed in all strains throughout the cultivation (Supplementary Figure S2). Within 16 days of cultivation, the strains expressing heterologous cellulases degraded 50%–70% of PASC (Figure 3). Strain A1 showed notably higher PASC degradation, although the differences in PASC degradation among the strains expressing heterologous cellulases were not statistically significant. This was likely due to relatively high experimental variability in determining the degradation, especially when higher amounts of PASC still remained.

### 3.4 Production of fermentation metabolites during growth on PASC

**FIGURE 4:**
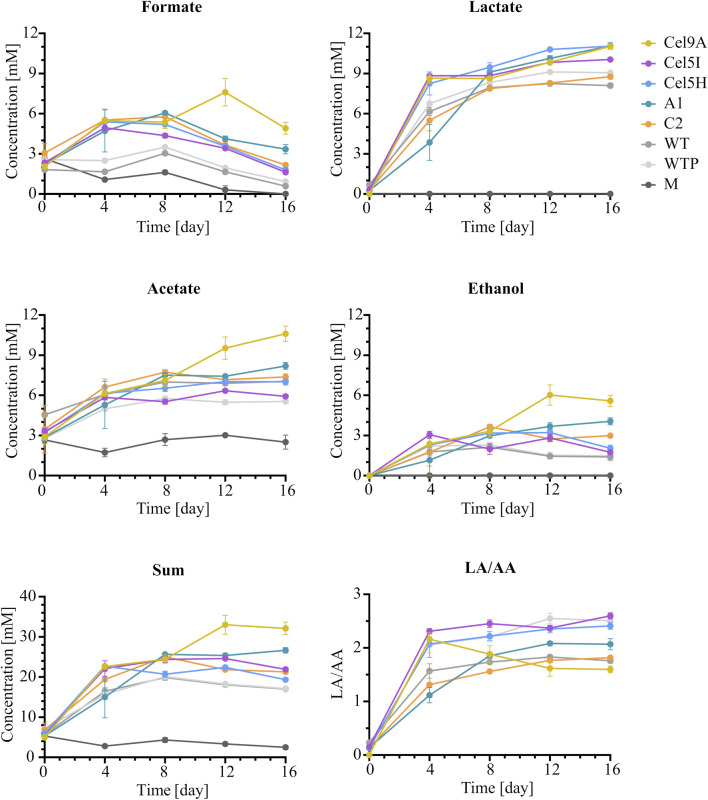
Production of fermentation metabolites during growth of genetically engineered *L. cremoris* strains on PASC. Concentrations of different metabolites (formate, lactate, acetate and ethanol) are shown individually or as the total sum of all four metabolites. LA/AA: ratio between the concentrations of lactic and acetic acid. Data are presented as mean ± SD from at least three biological replicates. A1: *L. cremoris* NZ9000 co-expressing *cel5H* and *cel9A* cellulase genes. C2: *L. cremoris* NZ9000 co-expressing *cel5H* and *cel5I* cellulase genes. All cellulase producing strains were grown in M17 medium supplemented with PASC. Controls: *L. cremoris* NZ9000 carrying an empty pNBBX plasmid grown in M17 medium (WT) or in M17 medium supplemented with PASC (WTP). M: uninoculated M17 medium supplemented with PASC.

During anaerobic growth of *L. cremoris* on PASC, mixed acid fermentation was observed. Apart from lactic acid, formate, acetate and ethanol were also detected in extracellular media (Figure 4). The extent of mixed acid fermentation was evaluated with lactic acid to acetic acid (LA/AA) ratio. Overall, the highest LA/AA ratio (indicating less mixed acid fermentation), with values above 2, was measured for strains Cel5I, Cel5H and WTP, reaching values above 2 already after 4 days of fermentation. The lowest LA/AA ratio (1.1) was measured for A1 strain (co-expressing Cel5H and Cel9A) on day 4, mainly due to lower LA production compared to the other strains. However, LA/AA ratio in A1 strain drastically increased after extended fermentation time, reaching values above 2 on day 12. In contrast, a decline in LA/AA ratio was observed for Cel9A strain, from around 2 on day 4, to around 1.6 on day 12; the latter value was the lowest LA/AA ratio among all strains after 12 days of culturing. Although the Cel9A strain was among the strains that produced the highest final amount of lactic acid, it also produced the highest amount of other fermentation products, indicating stronger mixed-acid fermentation, and thus the lowest LA/AA ratio. In the Cel9A strain, mixed-acid fermentation was the most pronounced, reflecting elevated energy demand and a metabolic shift toward acetate synthesis coupled to ATP formation by acetate kinase. Of note, cellulose hydrolysis by Cel9A resulted in a slow release of cellodextrins (Figure 2B), most of which are poorly metabolized by the strain (Supplementary Figures S3, S4), As a consequence the growth of the Cel9A strain under these conditions was essentially carbon-limited. Such limitation imposes selective pressure on cells to maximize ATP production per mole of available sugar, thereby promoting mixed acid fermentation. In contrast, the cellulases Cel5H and Cel5I enabled a more rapid release of larger amounts of sugars (Figure 2B) that are readily utilized by the strain (Supplementary Figures S3, S4). Under these less carbon-limited conditions, the cells experienced lower pressure to maximize ATP yield per mole of substrate, resulting in less pronounced mixed acid fermentation, as evident by higher LA/AA ratio. At the end of cultivation, all strains expressing heterologous cellulases (except C2, co-expressing Cel5H and Cel5I) produced comparable amounts of lactic acid (around 1 g/L). These were 20%–30% higher than that produced by wild type strain.

## 4 Discussion

Recently, it has been shown that *L. cremoris* NZ9000 producing a single heterologous cellulase (Cel9A, Cel5I or Cel5H) grows on insoluble cellulose PASC (Štravs et al., 2025). To improve degradation of cellulose by *L. cremoris*, we attempted in the present study to establish co-production of two cellulases (selected from Cel9A, Cel5H, Cel5I) in a single strain. Individual genes encoding cellulases were previously cloned under constitutive promoter in *L. cremoris*; however, we were unable to clone two cellulase genes under separate constitutive promoters. It was already reported that cellulases might be toxic for host organism when overproduced heterologously, making construction of cellulase co-expression system unsuccessful (Mingardon et al., 2011; Liu et al., 2019). Difficulties in developing recombinant strains that co-produce two cellulases, often caused by deleterious effects and the high metabolic burden imposed on the cells, can be bypassed by the establishment of co-cultures composed of two or more strains, each producing a single cellulase or cellulosomal component (Liu et al., 2019; Moraïs et al., 2014; Stern et al., 2018; Wan et al., 2024; Ma et al., 2025). Although co-cultures can serve as a practical alternative at laboratory scale, their implementation and management at larger industrial scales are considerably more complex. Therefore, the development of a single strain capable of secreting multiple cellulases remains a preferable objective for establishing consolidated bioprocesses. To overcome this problem, we cloned one of the cellulase-encoding gene under an inducible promoter, while keeping the other under the constitutive promoter. Alternatively, both genes were cloned under separate inducible promoters (Figure 1A). With this approach, we succeeded in co-production of different cellulase pairs in *L. cremoris* cells. To date, only a few studies reported successful co-production of two cellulases in LAB (Gandini et al., 2017; Guo et al., 2019; Wan et al., 2024). These studies employed bicistronic expression of cellulase genes from a single promoter, which makes the system easier to design. However, co-expression of genes from multiple cassettes enables better regulation of production of individual proteins (Kim et al., 2004; Berlec et al., 2018; Liu et al., 2026), which in this case is important for obtaining an optimal ratio between the produced enzymes and consequentially, enhanced synergistic effects on degradation of cellulosic substrate.

The highest cellulolytic activity on PASC was observed in culture supernatants of strains A1 (cellulase pair Cel5H-Cel9A) and C2 (cellulase pair Cel5I-Cel5H) (Figure 2B). The activity on microcrystalline cellulose Avicel was substantially lower than on PASC, regardless of whether cellulases were co-produced in a single strain or co-produced by coculturing two strains, each producing a single cellulase. The activity measurements of cellulases from culture medium on PASC indicated potential synergism between the cellulases for strains A1 (cellulase pair Cel5H-Cel9A) and C2 (cellulase pair Cel5I-Cel5H) (Supplementary Figure S1). This was in line with the highest PASC-degrading activities detected in culture supernatants of A1 and C2 strains, and indicates that synergism plays an important role in achieving higher overall activity of co-produced cellulases. Similarly, synergism between Cel5H and Cel9A or Cel5H and Cel5I was also suggested when these cellulase pairs were produced in co-cultures. In such systems, cell resources are divided either among different strains (e.g., nutrient availability) or within the same strain among multiple overexpressed proteins (e.g., limitations in transcriptional, translational, biosynthetic, and secretory machinery, as well as building blocks availability). Consequently, the amount of each individual recombinant protein in monoculture (when produced alone) is typically higher than when it is co-produced with another protein or produced in a co-culture system. As a result, the total cellulolytic activity of cells co-producing two cellulases is generally lower in the absence of synergism than the activity of the single most active cellulase expressed individually; this was also observed in previous studies (Wan et al., 2024). Previous study reported no synergism in activity among equimolar ratios of purified recombinant cellulases Cel9A, Cel5H and Cel5I on microcrystalline cellulose Avicel (David et al., 2025). In contrast, we observed that the cellulase pairs Cel5H-Cel9A and Cel5H-Cel5I, present in dialyzed culture media, displayed potential synergistic effects on amorphous cellulose PASC (Supplementary Figure S1). This suggests that this synergy depended on the quantity and ratio of each cellulase in the combination. It is well known that synergism between certain cellulases could differ on different cellulosic substrates (Andersen et al., 2008; Kostylev and Wilson, 2012). Furthermore, several studies demonstrated that mixtures of free, non-complexed cellulases often appear to achieve the highest cellulase synergy at non-equimolar ratios (Jeoh et al., 2006; Zohu and Ingram, 2000; Yang et al., 2016; Mazzoli, 2012). This is similar to the actual conditions in naturally occurring cellulolytic microorganisms, where different cellulases are expressed at different levels.

Although *L. cremoris* strains are primarily isolated from dairy environments, they possess many genes related to plant sugar utilization, indicating their ancestors may have plant origin (Siezen et al., 2011; Price et al., 2012). *Lactococcus cremoris* MG1363 has three different predicted transporters (PtC, CelB and llmg_1244) that presumably allow cellobiose transport into the cell (Linares et al., 2010; Solopova et al., 2012; Solopova et al., 2017). All three transporters are component of phosphotransferase system (PTS) and are subject to catabolite repression. CelB and llmg_1244 are inactive, and can become activated only when *L. cremoris* encounters a plant sugar–rich environment (Solopova et al., 2012; Solopova et al., 2017). Although in theory, the *L. cremoris* MG1363 and NZ9000 strains differ only in the nisin-control genes *nisR nisK* integrated into the *pepN* gene, a separate laboratory evolution of these two strains led to further differences, including the ability of NZ9000 strain to metabolize cellobiose without catabolite repression. This is due to the two mutations in cre site (the binding site of carbon catabolite control protein A (CcpA)) in the promoter regions of *ptcC* gene (encoding cellobiose-specific PTS IIC component - cellobiose transporter) and *bglA* gene (encoding phospho-β-glucosidase) (Linares et al., 2010). Here, we demonstrate that NZ9000 strain grows on cellotriose at levels comparable to those on cellobiose and glucose, a capability has not been previously reported (Supplementary Figure S4). This explains the growth of cellulase-expressing *L. cremoris* NZ9000 on PASC observed in the present study and in the study by Štravs et al. (2025), despite the fact that the cellulases used produced substantial amounts of cellotriose in addition to cellobiose (Supplementary Figure S3). The mechanism of cellotriose metabolism in *L. cremoris* strain is not known yet. According to CAZy database, *L. cremoris* NZ9000 has 78 carbohydrate-active enzymes, of which 41 are glycoside hydrolases. Among them, there are two putative endoglucanases belonging to CH5 and GH8 family and ten (phospho)beta-glucosidases belonging to GH1 and GH3 family which might be involved in cellotriose hydrolysis to single glucose units. Besides, many beta-glucosidases found in nature can hydrolyze not only cellobiose, but also longer soluble cellodextrins such as cellotriose, thereby acting on a wider range of substrates (Tanaka et al., 2011; Del Pozo et al., 2012; Singh et al., 2016).

Although strain A1 produced cellulase combination with highest activity on PASC (Figure 2B) and on average degraded the greatest amount of PASC in anaerobic culture (Figure 3), it produced similar amount of lactic acid (Figure 4) and had similar final viable cell count (Figure 3) as the strain producing a single cellulase Cel9A [previously reported as the strain with the fastest growth on PASC (Štravs et al., 2025)]. Overproduction of two recombinant proteins consumes more cellular resources (ribosomes, ATP, amino acids…) than overproduction of one recombinant protein, which could explain the lack of higher yields of metabolic acids with the A1 strain. Growth under anaerobic conditions led to less pronounced mixed-acid fermentation than the growth reported previously under microaerophilic conditions (Štravs et al., 2025). In the present study, the production of formate, ethanol and acetate was lower, resulting in at least 3 to 5 times higher LA/AA ratio (Figure 4), compared to the microaerophilic conditions (Štravs et al., 2025). Also, more lactic acid was produced in the same period of time; in the first 4 days of cultivation under anaerobic conditions, the production of lactic acid was up to two times higher than that previously reported under microaerophilic conditions (Štravs et al., 2025). It is known that the presence of oxygen and the type of main carbon source influence the shift from homolactic to mixed-acid fermentation, as well as the distribution of fermentation end-products (lactate, formate, acetate, ethanol, etc.) (Neves et al., 2005; Chan, 2014; Douwenga et al., 2023). Previous studies have shown that carbon distribution among metabolic products and biomass in microorganisms varies depending on the glucose oligomers or polymers utilized (Desvaux, 2006). This is in line with our observation that strains that express different heterologous cellulases produce not only different cellulose degradation products, but also different fermentation end products (Figure 4). Longer cultivation under anaerobic conditions in this study also enabled more extensive degradation of PASC (Figure 3) compared with previously reported cultivation under microaerophilic conditions (Štravs et al., 2025). This also shows that cellulases Cel9A, Cel5I and Cel5H remain active and could be used for longer cultivation periods.

In this study we achieved the co-production of two cellulases from separate expression cassettes in *L. cremoris*. This strategy enables independent modulation of each cellulase, allowing their production levels to be optimized to enhance synergy, and consequently improve the cellulolytic ability of the engineered bacteria. However, at the current stage of development, the engineered strains are able to grow independently only on amorphous cellulose; therefore, additional pretreatment of lignocellulosic biomass (chemical, physical, or physicochemical) would be required to improve cellulose accessibility (Zhao et al., 2022). To further enhance the synergy and overall cellulolytic activity, the development of a minicellulosome-based enzyme complex in a single strain could represent a promising strategy for advancing genetically engineered lactic acid bacteria with cellulolytic capabilities. In cellulosomes, enhanced synergy between cellulases arises from the proximity of enzymes in the complex, which facilitates cooperative interactions (Moraïs et al., 2014; David et al., 2025). Enhanced performance may be obtained also through the inclusion of cellulases that efficiently degrade crystalline cellulose and act synergistically with the currently employed cellulases. Moreover, the A1 strain developed in this study, which exhibits the highest PASC degradation, could be further optimized through laboratory adaptive evolution to enhance cellulase secretion rates and improve the utilization of cellulose degradation products. Additionally, to improve the production of target metabolites of cellulose fermentation, the impact of individual cellulose degradation products (soluble cellodextrins of different lengths) should be investigated. This could eventually help to select the cellulases for co-expression regarding the profile of their degradation products and interventions in metabolic pathways to promote the production of target metabolites. Nevertheless, further improvement of the designed strain for potential use in consolidated bioprocessing could also include co-expression of additional enzymes, such as xylanases, to more efficiently exploit the full carbon potential of lignocellulosic biomass.

## Data Availability

The original contributions presented in the study are included in the article/Supplementary Material, further inquiries can be directed to the corresponding author.
