# Supplementary material for: Engineering Lactococcus cremoris strains co-expressing two cellulase genes for growth on cellulose
Source: Front Bioeng Biotechnol. 2026 Jun 17;14:1857763. doi: 10.3389/fbioe.2026.1857763 (PMC13318888; doi:10.3389/fbioe.2026.1857763)
Supplement: Supplementary file 1 [file DataSheet1.pdf]

Supplementary Material

1 Supplementary Figures and Tables

Table S1: Plasmids, primers and strains used in this study. All plasmids contain gene for chloramphenicol resistance encoding chloramphenicol acetyltransferase. PepN: constitutive promoter; PnisA: nisin-inducible promoter. spUsp45: secretion signal of the Usp45; TT: transcription terminator. The F and R letters in primer names refer to forward and reverse primers, respectively.

**Strain**	**Genotype**		**Reference**
L. cremoris NZ9000	MG1363; *pepN*::*nisRK*		NIZO
L. cremoris NZ3900	MG5267 derivative (strain MG1363 containing lac operon in the chromosome); Δ*lacF*, *pepN*::*nisRK*		(De Ruyter et al., 1996a; Van Rooijen et al., 1992)
**Plasmid**	**Features**		**Reference**
pNZ8148			
pNBBX	pNZ8148 containing NheI, BglII, BclI and XhoI restriction sites		(Plavec et. al., 2021)
pPepN_sp_Cel9A	pNZ8148 containing gene fusion of sp_{Usp45} and *cel9A*; P_{nisA} promoter is replaced with P_{pepN} promoter		(Štravs et. al., 2025)
pPepN_sp_Cel5H	pNZ8148 containing gene fusion of sp_{Usp45} and *cel5H*; P_{nisA} promoter is replaced with P_{pepN} promoter		(Štravs et. al., 2025)
pPepN_sp_Cel5I	pNZ8148 containing gene fusion of sp_{Usp45} and *cel5I*; P_{nisA} promoter is replaced with P_{pepN} promoter		(Štravs et. al., 2025)
pPnisA_sp_Cel9A	pNZ8148 containing gene fusion of sp_{Usp45} and *cel9A*		This study
pPnisA_sp_Cel5H	pNZ8148 containing gene fusion of sp_{Usp45} and *cel5H*		This study
pPnisA_sp_Cel5I	pNZ8148 containing gene fusion of sp_{Usp45} and *cel5I*		This study
pNBBX_PnisA_sp_Cel9A	pNBBX containing expression cassette PnisA_sp_Cel9A_TT		This study
pNBBX_PnisA_sp_Cel5H	pNBBX containing expression cassette PnisA_sp_Cel5H_TT		This study
pNBBX_PnisA_sp_Cel5I	pNBBX containing expression cassette PnisA_sp_Cel5I_TT		This study
pNBBX_PepN_sp_Cel9A	pNBBX containing expression cassette PepN_sp_Cel9A_TT		This study
pNBBX_PepN_sp_Cel5H	pNBBX containing expression cassette PepN_sp_Cel5H_TT		This study
pNBBX_PepN_sp_Cel5I	pNBBX containing expression cassette PepN_sp_Cel5I_TT		This study
A1	pNBBX containing expression cassettes PnisA_sp_Cel5H_TT and PnisA_sp_Cel9A_TT		This study
A2	pNBBX containing expression cassettes PepN_sp_Cel5H_TT and PnisA_sp_Cel9A_TT		This study
B1	pNBBX containing expression cassettes PnisA_sp_Cel5I_TT and PnisA_sp_Cel9A_TT		This study

B2	pNBBX containing expression cassettes PepN_sp_Cel5I_TT and PnisA_sp_Cel9A_TT		This study
C1	pNBBX containing expression cassettes PnisA_sp_Cel5I_TT and PnisA_sp_Cel5H_TT		This study
C2	pNBBX containing expression cassettes PepN_sp_Cel5I_TT and PnisA_sp_Cel5H_TT		This study
**Primer**	**Sequence**	**Amplification**	**Reference**
Cel5H_B2_Bam	5'-ATAAGGATCCGCAGATGTTGCACCTTTAACCG -3'	*cel5H*	(Štravs et. al., 2025)
Cel5H-R-Xba	5'-ATTTTCTAGATTACCAGCTACCAAATTGCAGGG -3'	*cel5H*	(Štravs et. al., 2025)
Cel9A-F-Bam	5'-ATAAGGATCCGCGGAAACCAATTATAATTACGGAGAAG -3'	*cel9A*	(Štravs et. al., 2025)
Cel9A-R-Hind	5'-ATTTAAGCTTTTATGGTTCGACTCCCCAAACC -3'	*cel9A*	(Štravs et. al., 2025)
Cel5I2_F_Bam	5'-ATAAGGATCCGCCGAACCTGATTCTTCAC -3'	*cel5I*	(Štravs et. al., 2025)
Cel5I2_R_Xba	5'-ATTTTCTAGATTAGTTCTCAAATCTGACATTATCTACATACA -3'	*cel5I*	(Štravs et. al., 2025)
pNZFor 3072	ATGAGATAATGCCGACTG	PnisA	
Pnis_R_NcoI	AAATCCATGGTGAGTGCCTCCTTATAATTTATTTTG	PnisA	This study
NB-F-PpepN	AAAAAAGCTAGCATATAGATCTCTGTAAAAGCTGTCA	BglBrick expression cassettes with PepN promoter	This study
BX-R-TT2	AAAAAACTCGAGATATTGATCAAACGATTATGCCGATAACTAAAC	BglBrick expression cassettes	(Plavec et. al., 2021)
NB-F-PnisA2	AAAAAAGCTAGCATATAGATCTAGTCTTATAACTATACTGAC	BglBrick expression cassettes with PnisA promoter	(Plavec et. al., 2021)

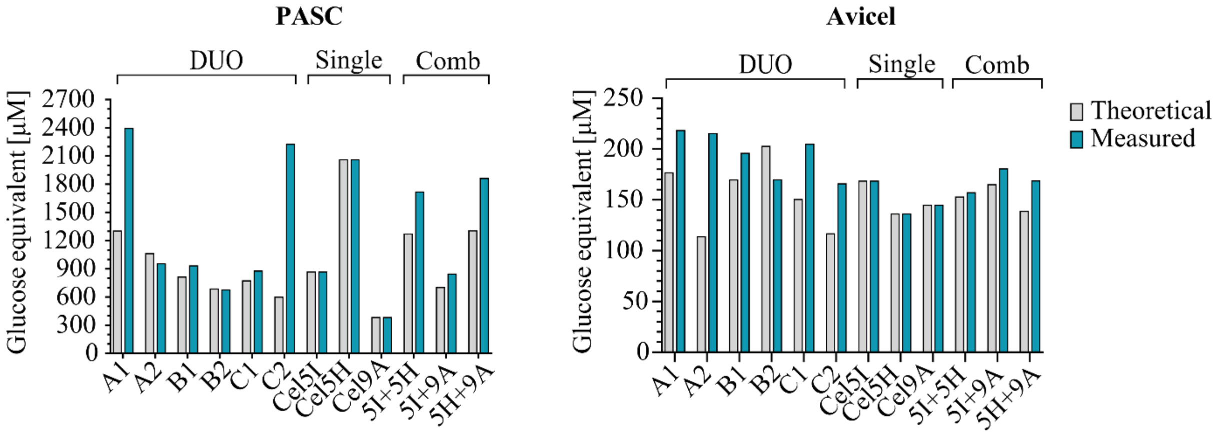

Figure S1: Theoretical and measured average activity of cellulases (present in dialyzed medium) on PASC and Avicel. DUO: medium of *L. cremoris* cells simultaneously expressing two cellulase genes (A1, A2, B1, B2, C1, C2). Comb: medium of *L. cremoris* cells co-cultures each expressing single cellulase gene (*cel5I*, *cel5H*, *cel9A*). Single: medium of *L. cremoris* cells expressing single cellulase gene (*cel5I*, *cel5H*, *cel9A*). Activity was measured spectrophotometrically by determining the amount of reducing sugars released and presented as glucose equivalents. Theoretical activity is the expected total activity considering no synergy between the cellulases, calculated by summing the measured activities of relevant cellulases, weighed by their quantity determined by densitometric analysis of SDS-PAGE (Fig. 1c) bands in Comb and DUO cultures.

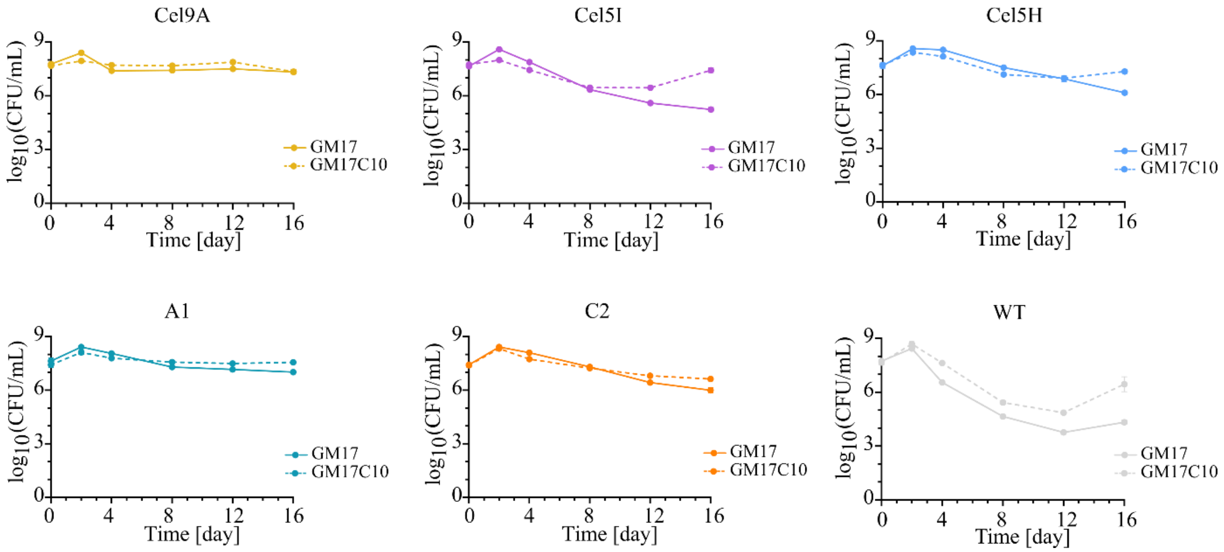

Figure S2: Plasmid segregational stability of *L. cremoris* strains grown on PASC. All cells (solid line): Viable *L. cremoris* cells in culture medium capable of forming colonies on GM17 agar plates without antibiotic. Cells with plasmid (dashed line): Viable *L. cremoris* cells in culture medium that contain plasmid and are capable of forming colonies on GM17 agar plates supplemented with 10 µg/mL chloramphenicol (GM17C10). *L. cremoris* NZ9000 strains expressing single cellulase gene (*cel9A*, *cel5H*, *cel5I*) or co-expressing two cellulase genes (A1, C2). A1: *L. cremoris* NZ9000 strain co-

expressing *cel5H* and *cel9A* cellulase gene. C2: *L. cremoris* NZ9000 strain co-expressing *cel5H* and *cel5I* cellulase gene. WT: *L. cremoris* NZ9000 carrying an empty pNBBX plasmid.

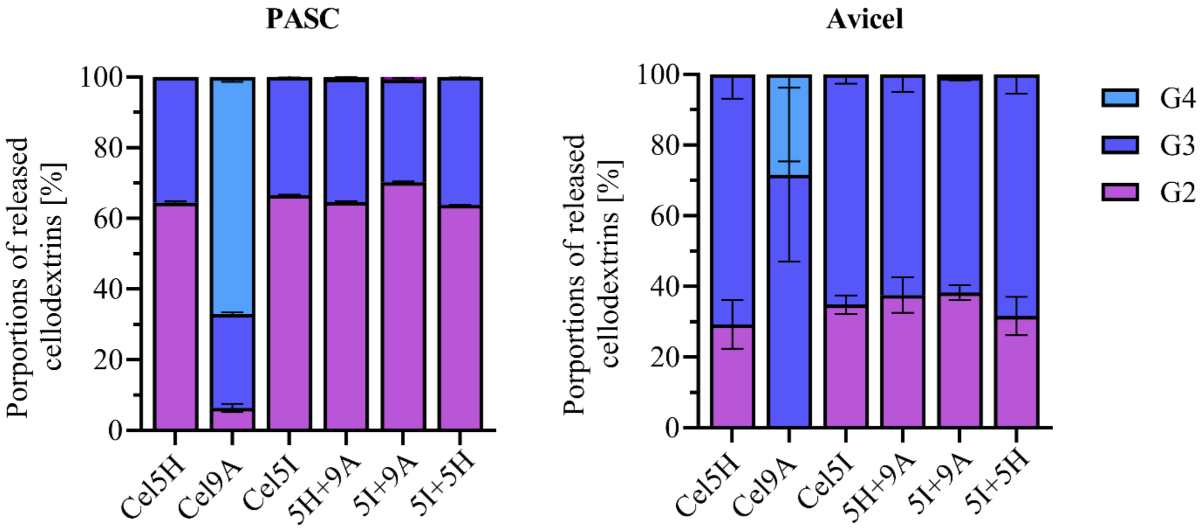

Figure S3: Proportions of cellodextrins released from PASC and Avicel by cellulases present in the dialyzed monoculture supernatants of *L. cremoris* strains or co-cultures expressing heterologous cellulases (Cel5H, Cel9A, Cel5I). Samples containing secreted cellulases for activity assays and activity assay were conducted as previously described (Štravs et al. 2025). Quantification of the released cellodextrins was performed with high-performance anion-exchange chromatography with pulsed amperometric detection (HPAEC-PAD) using a Dionex ICS 3000 system (Sunnyvale) with (4 × 250 mm) CarboPac PA1 column using a formerly described procedure (Ravachol et al., 2014). G2: cellobiose, G3: cellotriose, and G4: cellotetraose. Data are presented as mean ± SD from three biological replicates.

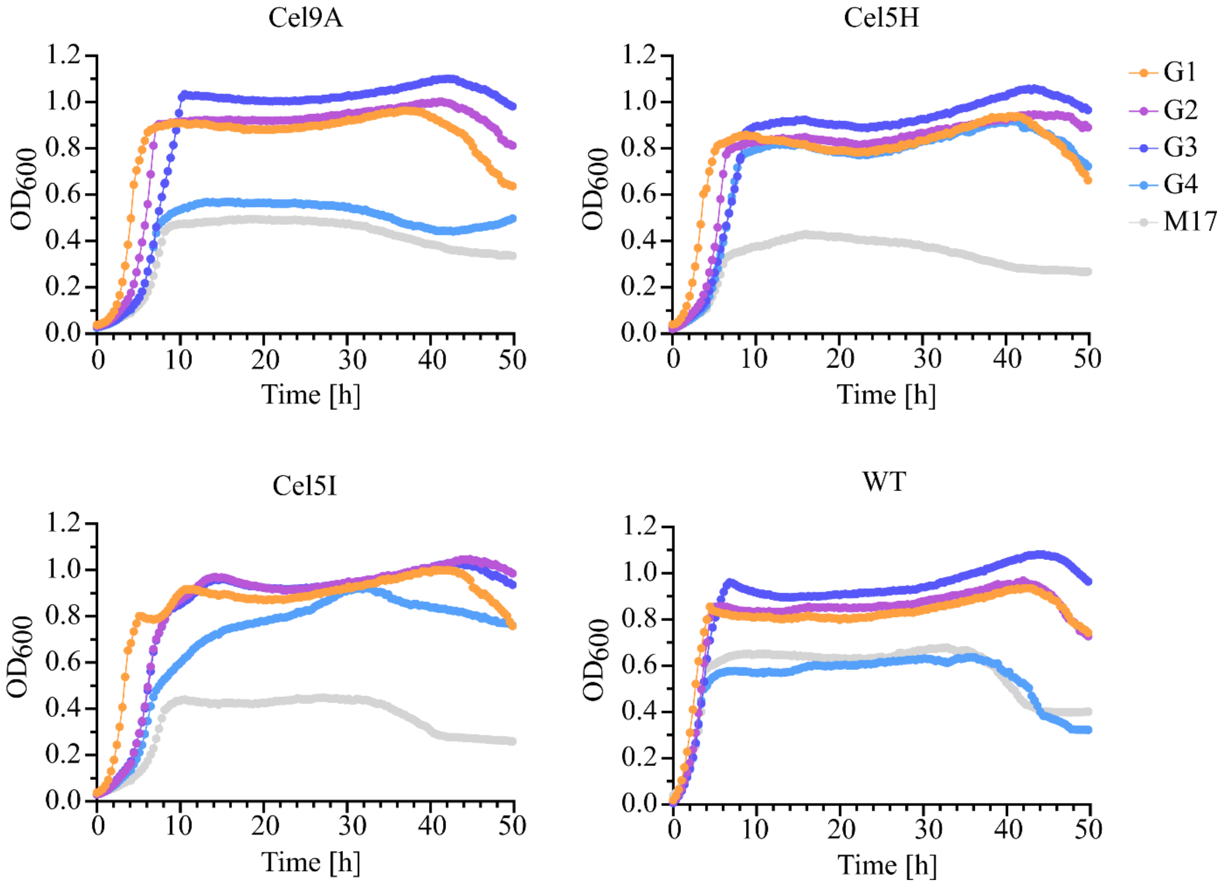

Figure S4: Growth of *L. cremoris* strains expressing cellulase gene (*cel5H*, *cel9A*, *cel5I*) or wild type (WT) on M17 medium supplemented with 2 g/L of sugar (G1: glucose, G2: cellobiose, G3: cellotriose and G4: cellotetraose) or not supplemented with any sugar (M17). Optical density (OD) at 600 nm was monitored on microplate reader throughout 50 h of growth.

2 References

De Ruyter, P. G. G. A., Kuipers, O. P., Beerthuyzen, M. M., Van Alen-Boerrigter, I., & De Vos, W. M. (1996). Functional analysis of promoters in the nisin gene cluster of *Lactococcus lactis*. *J. Bacteriol*, 178, 3434–3439. https://doi.org/10.1128/JB.178.12.3434-3439.1996

Plavec, T. V., Ključevšek, T., and Berlec, A. (2021). Introduction of modified BglBrick system in *Lactococcus lactis* for straightforward assembly of multiple gene cassettes. *Front. Bioeng. Biotechnol.* 9:797521. doi: 10.3389/fbioe.2021.797521

Ravachol, J., Borne, R., Tardif, C., de Philip, P., & Fierobe, H. P. (2014). Characterization of all family-9 glycoside hydrolases synthesized by the cellulosome-producing bacterium *Clostridium cellulolyticum*. *J. Biol. Chem*., 289, 7335–7348.

Štravs, P., David, H., Fierobe, H. P., Perret, S., and Berlec, A. (2025). Development of cellulose-degrading lactic acid bacterium *Lactococcus cremoris* by genetic engineering. *Bioresour. Technol.* 438:133177. doi: 10.1016/j.biortech.2025.133177

Van Rooijen, R. J., Gasson, M. J., & De Vos, W. M. (1992). Characterization of the *Lactococcus lactis* lactose operon promoter: Contribution of flanking sequences and LacR repressor to promoter activity. *J. Bacteriol*, 174, 2273–2280. https://doi.org/10.1128/JB.174.7.2273-2280.1992
